# The Effect of Dietary Humic Preparations on the Content of Essential and Non-Essential Chemical Elements in Hen Eggs

**DOI:** 10.3390/ani10081252

**Published:** 2020-07-23

**Authors:** Zbigniew Dobrzański, Katarzyna Chojnacka, Tadeusz Trziszka, Sebastian Opaliński, Łukasz Bobak, Damian Konkol, Mariusz Korczyński

**Affiliations:** 1 Department of Environment Hygiene and Animal Welfare, Faculty of Biology and Animal Sciences, Wroclaw University of Environmental and Life Sciences, Chełmońskiego 38C, 51-630 Wroclaw, Poland; zbigniew.dobrzanski@upwr.edu.pl (Z.D.); sebastian.opalinski@upwr.edu.pl (S.O.); damian.konkol@upwr.edu.pl (D.K.); 2 Department of Advanced Material Technologies, Wroclaw University of Science and Technology, Smoluchowskiego 25, 50-372 Wroclaw, Poland; katarzyna.chojnacka@pwr.edu.pl; 3 Department of Functional Food Products Development, Wroclaw University of Environmental and Life Sciences, Chełmońskiego 37, 51-630 Wroclaw, Poland; tadeusz.trziszka@upwr.edu.pl (T.T.); lukasz.bobak@upwr.edu.pl (Ł.B.)

**Keywords:** dietary humic preparations, laying hens, egg albumen, egg yolk, chemical composition

## Abstract

**Simple Summary:**

In addition to protein, fatty acids and vitamins, hen eggs also contain many minerals, including macroelements, microelements and trace elements. Currently, many different organic mineral supplements are introduced into the diet of laying hens, which can affect the content of chemical elements (essential and non-essential) in the albumen and yolk. These effects are not fully understood. In the present work, the effect of the addition of humic preparations to the standard feed mixture on the content of essential and non-essential chemical elements in albumen and yolk of hen eggs was assessed. The obtained results indicate that only some elements significantly increase in the albumen and yolk when more of them are in a feed mixture enriched with humic preparations.

**Abstract:**

This study was conducted to determine the effect of dietary supplementation with two humic preparations, Humokarbowit (HKW) and Humobentofet (HBF), on the mineral content of the albumen and egg yolk of Lohmann Brown hens. The content of macroelements (Ca, K, Mg, Na, P, S), microelements (Al, Ba, Cu, Fe, I, Mn, Si, Sr, Zn) and trace elements (Ag, As, Be, Bi, Cd, Co, Cr, Ga, Hg, Li, Mo, Ni, Pb, Rb, Sb, Se, Sn, Ti, Tl, V, W, Y and Zr) in the feed mixture (FM), albumen and yolk were presented. The material was collected from laying hens kept in a cage system in two groups, control (C) and enriched (E), with standard feed and feed enriched with humic preparations, respectively. The enriched feed mixture was characterised by a significantly higher Ag, Ba, Be, Bi, Co, Fe, Ga, Hg, K, Mg, Ni, S, Sb, Si, Zn and Zr content compared to the standard, basal mixture. Only some of these elements were found in significantly increased levels in albumen (Bi, Co, Ni, S) and yolk (Bi, Fe, K, Sb). Another noteworthy finding was a significantly lower concentration of Na in the content of eggs from the E-Group, which corresponds to the content of this important macronutrient in the feed. In addition, a significant increase in the concentration of elements such as Al, I, Li, Sr, Ti, Tl, Y, W was noted with a reduction in Cd, Cr, Hg, Mn, Rb, Sn in Group-E, which indicates a complicated egg formation processes, including biotransfer-essential and non-essential chemical elements.

## 1. Introduction

Hen eggs are an important component of the human diet. Their physico-chemical and biological properties are quite well known, and in recent years their role as a source of nutraceutical and biomedical substances has increased [1,2,3,4]. In addition to protein, fats (fatty acids), enzymes and vitamins, egg albumen and yolk also contains minerals (macroelements, microelements, trace elements) [5,6,7,8,9]. While the composition and content of organic substances in eggs is quite well known, the mineral composition and the possibility of its modification is the subject of few studies.

It is known that hen eggs are a good source of iron (Fe), phosphorus (P) and sulphur (S). The eggs also provide calcium (Ca), chlorine (Cl), potassium (K) magnesium (Mg) and sodium (Na). Smaller amounts of ions include: silver (Ag), aluminium (Al), boron (B), barium (Ba), bromine (Br), cobalt (Co), chromium (Cr), copper (Cu), fluorine (F), iodine (I), lithium (Li), manganese (Mn), molybdenum (Mo), rubidium (Rb), selenium (Se), silicon (Si), strontium (Sr), titanium (Ti), vanadium (V), uranium (U) and zinc (Zn). Eggs may also contain heavy metals such as arsenic (As), bismuth (Bi), cadmium (Cd), mercury (Hg), lead (Pb), thallium (Tl) and others in trace concentrations [5,10,11]. Rankin [12] classifies the following elements as essential for humans and animals: Ca, Cl, K, Mg, Na, P, S (macronutrients) and Cr, Co, Cu, F, Fe, I, Mn, Mo, Ni, Se, Zn, (As), (B), (Ba), (Si), (Sn), (V), (W) (micronutrients)—the elements enclosed in brackets are possible but unconfirmed. The following elements are included in the non-essential group: Ag, Al, Au, Be, Bi, Br, Cd, Cr, Cs, Hf, Hg, In, Li, Nb, Rb, Re, Pb, Sb, Sc, Sr, Ta, Te Ti, Tl, Zr and platinum group metals (Ir, Os, Rh, Ru, Pd, Pt).

Metals are ubiquitous in the environment and they can easily accumulate in biological organisms including plants and animals, in animal origin products such as eggs, milk, and meat. Among the 35 naturally existing metals, 23 possess high specific density above 5 g/cm^3^ with atomic weights greater than 40.04 and are generally termed heavy metals. Theses metals include: Ag, Au, Bi, Ce, Cd, Cr, Co, Cu, Fe, Ga, Hg, Mn, Ni, Pb, Pt, Sb, Sn, Te, Tl, U, V and Zn. Some of these heavy metals such as Co, Cr, Cu, Mg, Fe, Mo, Mn, Ni, Se and Zn are essential nutrients that are required for various physiological and biochemical functions in the body, but may cause acute or chronic toxicities in large doses [13].

A slightly different classification of elements is given by Lim and Schoenung [14]. Heavy metals include Ag, As, Ba, Be, Cd, Cr, Co, Cu, Hg, Mo, Ni, Pb, Sb, Se, V and Zn, with Ag, Ba, Be, Mo and V having potentially toxic properties. It is interesting that all these elements were found in the content of eggs, albumen and yolks, though mainly in trace amounts. To make the problem more complicated, it should be noted that some heavy metals also perform important biological functions in animals and humans, and are even necessary in maintaining various physiological processes. These include Co, Cr, Cu, Fe, Mn, Mg, Mo, Se and Zn. In addition, some such as Ag, As, Au, Bi, Cu, Fe, Ru, Pt and Zn have therapeutic significance and are sometimes used in the treatment of disease. Cadmium, Hg and Pb are the most harmful because they have toxic and carcinogenic properties [14]. Other authors include three other heavy metals in the last four: Al, Cr and Fe [15]. In contrast, International Agency for Research on Cancer [16] published lists of human carcinogens and toxic substances. These lists include As, Be, Cd, Co, Cr, Hg, Ga, In, Ni, Pb and V. It is obvious that the action of these elements depends on the chemical form and the dose (exposure) consumed, as well as their bioavailability. As discussed earlier, some heavy metals are limited in raw materials and feed for farm animals, as well as in foods of mineral, plant and animal origin. It is worth adding that the American Institute of Medicine [17] has developed dietary reference intakes (DRI) for 12 elements, including heavy metals (As, B, Cr, Cu, I, Fe, Mn, Mo, Ni, Si, V and Zn). In contrast, the Polish National Food and Nutrition Institute [18] developed standards for Ca, Cu, Fe I, F, Mg, Mn, P, Se and Zn daily demand levels (recommended dietary allowance—RDA), as well as standards for water and electrolytes (Na, K and Cl) at a level of sufficient intake (AI—adequate intake). Calculation of DRI or RDA values for specific egg consumption in the USA or Poland may be the subject of further work.

The mineral composition of hen eggs is genetically determined, but environmental (management) and nutritional (chemical composition of diet) factors can modify it to some extent [19,20,21,22,23]. For example, when examining the differences in the composition of hen eggs from four selected breeds, the highest content of Mg, Cu and Se was observed in eggs from Sussex hens, while Rhode Island Red hens had increased amounts of K, Fe and Mn. Eggs from Polish, Greenleg Partridge hens had increased concentrations of Zn, while Yellowleg Partridge eggs had increased levels of Ca and Na compared to the previously mentioned breeds of hens [20]. Other authors determined the concentration of eleven micronutrients and trace elements (Ba, Cu, Fe, Mn, Ni, Pb, Rb, Se, Sr, V, Zn) in hen egg samples taken from various poultry housing systems in Latvia (large-scale poultry farms, organic farm systems and households). Egg samples from organic farms had the most variable concentration range and the highest element content, while egg samples from other farming systems contained lower element levels [22]. American researchers [23] examined the impact of non-nutritional factors on mineral content of eggs and specifically analysed the effect of conventional battery cages, enrichable cage systems, enriched colony housing, and cage-free and free-range rearing systems on mineral concentrations of whole eggs from Tetra White and Hy-Line Brown hens at different ages. The authors observed that differences in egg mineral content were relatively small and were unlikely to have a substantial impact on human nutrition.

Evidence suggests that the mineral composition of feed mixtures, the origin and quality of nutrients and their chemical form and bioavailability have the greatest importance in the accumulation of essential and non-essential chemical elements in the hen’s eggs [11,24,25,26,27]. In recent years, many innovative feed additives have been used in laying hen nutrition. These additives include, among others, mineral preparations including humic and aluminosilicate raw materials [28,29,30,31,32]. It is not known, however, whether they affect the chemical composition of eggs in intensive farming of laying hens, and if so, to what extent.

The aim of this study was to determine the effect of a feed mixture enriched with humic preparations on the concentration of essential and non-essential elements in the albumen and yolk of Lohmann Brown hen eggs.

## 2. Materials and Methods

### 2.1. Ethical Statement

The feeding experiment on laying hens was approved by the Second Local Ethical Committee on Animal Testing at Wroclaw University of Environmental and Life Sciences (UPWr (No 17/2009, 09 February 2009).

### 2.2. Animal Material and Layout of the Experiment

A total of 60 Lohmann Brown (LB) laying hens were used in the experiment. Birds were housed in battery cages in a vivarium with a controlled climate and light programme (16L: 8D) located at the research station of UPWr in Swojec (Poland). Hens were put into cages (750 cm^2^ per hen) at 20 weeks of age and kept until the peak of laying (33 weeks of life). The feeding experiment was conducted for 90 days. The basal diet was formulated according to the nutrient recommendations for LB laying hens [33]. The laying hens were fed ad libitum and water was provided by two nipple drinkers separately for each cage. The birds were divided into two equal groups (30 hens in each group). The groups were divided into 6 replications (cages), with each replication consisting of 5 birds. Group-C received the standard feed mixture and Group-E received the standard feed mixture with the addition of 2.9% humic preparations (1.70% Humokarbowit (HKW) and 1.20% Humobentofet (HBF)), which were previously introduced into feed mixture (thorough mixing). These preparations are patented in Poland and are composed of peat, humodetrynite, bentonite, dolomite and plant oil. Both mixtures were sent to the Department of Animal Nutrition and Feed Management laboratory of the UPWr in order to determine their basic parameters (dry matter, crude ash, crude protein, crude fibre and crude fat) according to standard methods [34].

### 2.3. Sample Collection for Analyses

Eggs for tests were collected at the peak of laying (approximately 95%) for 5 consecutive days from all 12 cages. Every day, 5 undamaged eggs with similar mass (62–66 g) were randomly selected from both groups (C and E). Then the eggs were broken and the albumen was separated from the yolk and stirred thoroughly in a glass vessel. Five samples of albumen and yolk were collected from each group (C and E) each day leading to 60 eggs being collected in total. The egg material was stored for 10 days in a cold storage room (+4 °C; humidity: 45%) before being transferred to the chemical laboratory at the Wroclaw University of Science and Technology. Additionally, samples of feed mixtures (FMs) were collected from both groups every two weeks in an amount of approximately 0.5 kg each (n = 5) for chemical analysis.

### 2.4. Multielemental Analysis

All used chemicals were of analytical grade. Eggs from the two groups were collected and analysed for their element content by inductively coupled plasma optical emission spectroscopy (ICP-OES and ICP-MS). The specified mass of biological samples (feed—0.5 g, albumen—1.5 g and yolk—1.5 g) was digested in Teflon vessels with 5 mL of concentrated supra pure grade HNO_3_ from Merck (Whitehouse Station, NJ, USA) in a microwave oven (Start D, Milestone Srl Sorisole, Italy). An inductively coupled plasma optical emission spectrometer with ultrasonic nebulizer (Varian VISTA- MPX ICP- OES, Victoria, Australia) and an inductively coupled plasma mass spectrometer ICP-MS (ICP-MS Varian UltraMass-700 Instrument) were used in the analysis of the content of essential (Ca, K, Mg, Na, P and S) and non-essential elements (Ag, Al, As, Ba, Be, Bi, Cd, Ga, I, Li, Ni, Pb, Rb, Sb, Sn, Si, Sr, Ti, Tl, V, W, Y and Zr) in the feed mixture and the egg content. The level of other elements (Co, Cr, Cu, Fe, I, Mn, Mo, Se and Zn) was determined by ICP-MS, while the content of the remaining elements by ICP-OES. Mercury (total content) was measured directly in raw materials using an AMA-254 mercury analyzer (Altec Ltd., Czech Republic). The analyses were carried out in the Multielemental Analyses Chemical Laboratory at the Wroclaw University of Science and Technology, which is accredited by International Laboratory Accreditation Cooperation/Mutual Recognition Arrangement and the Polish Centre for Accreditation (nr AB 696), according to PN-EN ISO/IEC 17025. The quality of analytical process was controlled with the certified reference material CRM 8415 EGG POWDER, NIST.

### 2.5. Statistical Methods

The analysis was carried out using Statistica ver. 13.1. The data were presented as the mean and standard error of mean (SEM). The normality of the distribution was assessed using the Shapiro−Wilk test. If the distribution was normal, a Student’s t-test for independent samples was performed. If the distribution was not normal, a Mann−Whitney U test was carried out. Effects were considered significant at a probability of *p* < 0.05 and *p* < 0.01.

## 3. Results

### 3.1. Feed Mixture

The results of the analyses of feed mixtures (Table 1) show some variation in the average content of basic nutrients, but generally were within the limits given by different authors [8,14,18,21]. The difference in energy content is noticeable, which is probably due to the fact that humic preparations contained a certain level of fat. Both mixtures (control and experimental) were iso-protein and iso-energetic and contained similar levels of the main elements, such as Ca and P, but differed significantly in the level of 17 other elements. The feed from Group-E was characterised by a significantly higher content of Ag, Ba, Bi, Co, Fe, Ga, Hg, K, Mg, Ni, S, Sb, Si, Zn and Zr and a lower concentration of Na compared to the feed from Group-C, which is shown in Table 2. Increased concentrations of macroelements and trace elements resulted from the use of 2.9% HKW and HBF preparations containing raw organic mineral materials, such as peat, humodetrynite, bentonite, dolomite and plant oil. The decrease in Na in Group-E probably results from the fact that the content of salt (NaCl) was lowered in this mix, which was due to the fact that humic preparations contained it. The concentrations of the other tested elements, such as Al, As, Cd, Cr, Cu, I, Mn, Mo, Li, Rb, Pb, Se, Sn, Sr, Ti, Tl, V, W and Y, did not differ significantly between Group-C and Group-E. It is worth adding that the mixtures used did not contain excess heavy metals, for which the limits in Poland as well as other EU countries are As—2.0; Cd—2.0; Hg—0.1; Pb—5.0 mg/kg FM [34].

### 3.2. Albumen and Yolk

The results of chemical analyses (macro-, microelements and trace elements) of the albumen and yolk from Group-C and Group-E are shown in Table 3 and Table 4.

#### 3.2.1. Content of Macroelements in Eggs

Among the six macroelements, the level of S and Na was the highest in the albumen, while P, S and Ca were highest in the yolk. The maximum value observed in the albumen was from S (2114 mg/kg wet wt) and in the yolk was P (6032 mg/kg wet wt). The concentration of macroelements such as Mg, K and S in Group-E significantly increased the concentration of S in the albumen and K in the yolk. However, the reduced Na concentration in the experimental feed mixture significantly reduced the level of this important macronutrient in egg content (albumen and yolk) in Group-E. Despite the differences in the concentration of these elements, they are within the ranges given by other authors [8,20,35,36], although detailed comparison is difficult due to frequent mineral composition obtained from dry matter of whole eggs (yolk and albumen combined).

#### 3.2.2. Content of Microelements in Eggs

Among the eight microelements, Al and Si were the highest in the egg albumen, while Fe, Si and Zn were the highest in the yolk. The maximum value observed in the albumen refers to Si (6.86 mg/kg wet wt) and to Fe (81.8 mg/kg wet wt) in the yolk. The increased concentration of microelements, such as Ba, Si and Zn, in Group-E did not significantly affect the accumulation of these elements in egg content. The significant increase in Al concentration and decrease in Mn in the albumen, and the increase in concentration of Fe, I and Sr in the egg yolk from Group-E compared to Group-C, were noted. In relation to Cu, Fe, I, Mn and Zn, the obtained results are only partially consistent with data from other authors [7,10,21,35], which is understandable when the material (hen eggs) comes from different sources (e.g., breed, feed, management system). It is also difficult to refer to other metals (Al, Ba, Si and Sr), as no other works were found for comparison.

#### 3.2.3. Content of Trace Elements in Eggs

Among the 23 trace elements tested, Ni, Rb and Ti were most abundant in the egg albumen, while Cr, Rb and Sn and were most abundant in the egg yolk. The most abundant element observed in the albumen and the yolk was Rb (1.41 and 0.864 mg/kg wet wt, respectively). The increased concentration in Group-E of trace elements, such as Ag, Be, Bi, Co, Ga, Hg, Ni, Sb and Zr, significantly influenced the accumulation of only Bi, Co and Ni in the albumen and Bi and Sb in the yolk. There was also a significant increase in Al, Tl and W in Group-E albumen and a decrease in Cd, Cr, Rb and Sb compared to Group-C. In the yolk, however, these relationships concern Li, Ti, Tl, Y and W (growth) and Hg and Sn (decrease). It is difficult to explain the significant increase in Tl in the content of eggs from Group-E when both FMs had almost identical concentrations of this toxic trace element. Thallium interacts with several elements, such as K, Rb and S, which may explain its accumulation in egg content, especially in the yolk [11,37]. Tungsten (W) behaved similarly in that there was more of it in the Group-E (albumen and yolk) despite the fact that the feed mixtures had almost identical content of this element. Tungsten is an antagonist of Mo and V, which makes interpretation of the results difficult [38,39].

The other elements in both groups were observed in similar concentrations: in the albumen, Ag, As, Be, Ga, Hg, Li, Mo, Pb, Se, Sn, Ti, V, Y and Zr; and in the yolk, Ag, As, Be, Co, Cd, Cr, Ga, Mo, Ni, Rb, Pb, Se, V and Zr. It is worth adding that the content of the most toxic heavy metals, such as As, Cd, Hg and Pb, did not differ significantly between groups E and C. Some authors [21,28] reported the levels of As, Cd, Co, Cr, Mo, Ni, V, Se and Ti separately in egg albumen and yolk, which are only partially consistent with the results of their own research.

## 4. Discussion

The obtained results can be compared with others despite being implemented in other management conditions of laying hens (deep litter system). The authors [28] used HKW and HBF preparations in the nutrition of Lohmann Brown hens (ad libitum administration). The dietary humic preparations significantly increased only Se and decreased Mo concentration in the egg albumen and increased Fe and Se content in the egg yolk. The preparations did not influence Cr, Co, Cu, I, Mn or Zn content in the eggs. These results only partially resemble those obtained in this study, for instance, they confirm the increase in Fe concentration in the yolk and the lack of influence on the Cu and Zn content in the albumen and the yolk in Group-E. Other studies have shown that the use of iodine yeast in laying hen (Hy-Line Brown) nutrition raises the level of iodine in egg yolks by 80-90% compared to the control group and increases the concentration of this element in the eggshell 3-fold [25]. Another paper presents results of research on the bioaccumulation of copper, manganese, iron and zinc in LB laying hen eggs receiving diets containing organic forms of Cu, Mn and Fe (as yeast). The application of Cu caused a significant increase in Cu concentration in the egg content and the shell in the group receiving organic Cu compared to copper sulphate. The introduction of organic forms of Fe and Mn (yeast) to the feed mixture did not cause any significant changes in the content of these metals in eggs. Organic forms of Cu, Fe and Mn did not result in any interactions with respect to Zn, although an antagonistic influence of Cu (organic Cu group) and synergistic of Mn (organic Mn group) in the egg content was observed [11].

In other studies, the diet of laying hens was supplemented with soybean meals enriched with Cr, Cu, Fe and Zn. The results showed that the use of this feed additive in the diet of laying hens (Hy-Line Brown) influenced the transfer of microelements to eggs, in particular at increased dosing. Eggs were biofortified with Fe, Zn, and Cu and, to a lesser extent, Cr. Microelements accumulated mainly in the albumen. Transfer of trace elements to eggs was not linearly dependent on the dose of biologically bound microelements in the diet [27]. This was also confirmed in studies by Santoso and Fenita [36], who examined the effect of the addition of katuk (*Sauropus androgynus*) leaf extract (SALE) on the production parameters of laying hens (Decalb Warren) and the quality and chemical composition of their eggs. The results showed that the SALE supplementation did not significantly affect levels of calcium, phosphorus, iron and potassium, even at high doses. The SALE contained 2330 mg calcium, 980 mg phosphorus and 35 mg iron in 1 kg.

The content of trace elements in hen eggs (and other domestic birds) was analysed by Nisianakis et al. [10]. Hens (Lohmann Brown) were housed in one farm in northern Greece and the fed a cereal and legume based diet which did not contain any vitamin or mineral supplements. The level of As, Cd, Co, Cr, Cu, Mo, Mn, Ni, Se, V, Ti and Zn were determined by ICP-MS. The content of Co, Cr, Cu, Ni, V, Se and Zn in yolks was lower, but As and Mo were higher compared with results from our investigations. The concentration of Cd and Mn was similar. The content in the albumen of As, Cd, Co, Ni, and Se was lower, but Cr, Cu, Mn, Mo, V and Zn were higher compared with our results (mean values of the C and E groups).

The results obtained in this study do not fully answer whether hen eggs could be enriched with various elements. Their biotransfer to the albumen and the yolk is limited, as some authors point out [8,21,23,27]. This problem is convoluted by complex relationships between chemical elements (agonistic, antagonistic and synergistic interactions), which hinder the unequivocal interpretation of results [40,41]. The biological roles of many microelements or trace elements are also not fully understood.

## 5. Conclusions

The addition of humic preparations to the standard feed mixture for laying hens increased the content of sixteen chemical elements (essential and non-essential) and reduced only the Na concentration. The effect of feeding this enriched mixture caused significant increases in elements such as Al, Bi, Co, Ni, S, Tl, W in the albumen and Sb, Bi, I, Fe, K, Sr, Li, Tl, Ti, W and Y in the yolk, while it significantly decreased concentrations of Sb, Cd, Cr, Mn, Rb (albumen), Hg and tin (yolk). Lower levels of Na in the experimental diet also significantly reduced the content of this important macronutrient in the egg content. From the point of view of the nutritional value of eggs, it is very important to obtain a significant increase in the content of Co, I, Fe, Ni, K, Si, S, W (essential elements), while ensuring heavy metals (toxic) such as As, Cd, Hg, Pb or Tl do not exceed the values given by various authors. A full assessment of the legitimacy of the administration of humic preparations to laying hen diets must take into account studies on the physical characteristics of the eggs (shell thickness and strength) and their organoleptic characteristics, which will be the subject of upcoming work of the authors.

## Figures and Tables

**Table 1:** Nutrient composition of feed mixtures from both groups.

Compound	Control Group (C)	Experimental Group (E) *
Dry matter (%)	90.30	90.41
Crude ash (%)	12.18	12.97
Crude fibre (%)	6.61	6.54
Crude fat (%)	3.93	4,03
Crude protein (%)	16.79	16.50
Metabolic energy (MJ/kg)	11.58	11.72

* Basal diet + inclusion of 1.70% Humokarbowit (HKW) (Polish Patent No 172908) and 1.20% Humobentofet (HBF) (Polish Patent No 182963), when lowering the content of the NaCl.

**Table 2:** The content of macroelements, microelements and trace elements in the feed mixture (mg/kg).

Element	Group-C	Group-E	SEM	*p*-Value
Macroelements				
Ca	33,860	36,820	1312.5	0.138 ^†^
K	7030 ^A^	7630 ^B^	118.0	0.002 ^†^
P	5810	6024	61.5	0.079 ^†^
Mg	2373 ^A^	2582 ^B^	40.4	0.001 ^†^
S	1760 ^A^	2116 ^B^	60.5	0.001 ^†^
Na	1398 ^A^	1186 ^B^	37.8	0.001 ^†^
Microelements				
Fe	215.4 ^a^	285.2 ^b^	14.55	0.012 ^‡^
Al	162.3	177.4	8.59	0.711 ^†^
Zn	109.4 ^a^	123.1 ^b^	3.01	0.012 ^†^
Mn	108.3	117.6	3.95	0.245 ^†^
Si	78.41 ^A^	95.80 ^B^	2.960	0.001 ^†^
Sr	15.80	16.45	0.411	0.493 ^†^
Cu	15.23	17.21	1.290	0.916 ^‡^
Ba	5.64 ^A^	6.36 ^B^	0.142	0.002 ^†^
I	2.36	1.78	0.131	0.122 ^†^
Trace elements				
Ti	3.44	3.28	0.440	0.872 ^†^
Ni	1.34 ^a^	1.66 ^b^	0.061	0.028 ^‡^
Li	1.28	1.62	0.041	0.806 ^‡^
Cr	0.79	1.14	0.092	0.054 ^†^
Rb	0.76	0.80	0.020	0.530 ^‡^
Mo	0.66	0.49	0.050	0.071 ^†^
V	0.47	0.53	0.231	0.772 ^‡^
Y	0.43	0.39	0.072	0.327 ^†^
Bi	0.48 ^A^	1.90 ^B^	0.238	0.001 ^†^
Se	0.29	0.28	0.022	0.809 ^†^
Co	0.26 ^A^	0.32 ^B^	0.011	0.001 ^†^
Be	0.23 ^A^	0.31 ^B^	0.010	0.001 ^†^
Ga	0.19 ^A^	0.25 ^B^	0.012	0.007 ^†^
Cd	0.13	0.12	0.004	0.008 ^†^
As	0.12	0.11	0.009	0.834 ^‡^
Zr	0.10 ^A^	0.16 ^B^	0.010	0.003 ^†^
Pb	0.060	0.062	0.005	0.530 ^‡^
Ag	0.0065 ^A^	0.0200 ^B^	0.00201	0.001 ^†^
Sb	0.0056 ^A^	0.0092 ^B^	0.00062	0.001 ^†^
Sn	0.0041	0.0038	0.00402	0.469 ^†^
Hg	0.0025 ^A^	0.0044 ^B^	0.00030	0.001 ^†^
W	0.0023	0.0020	0.00026	0.668 ^†^
Tl	0.0008	0.0009	0.00011	0.391 ^‡^

SEM—standard error of mean; ^a,b^—significance of differences on the level *p* < 0.05; ^A,B^—significance of differences on the level *p* < 0.01; ^†^—based on t-test; ^‡^—based on Mann−Whitney U test.

**Table 3:** The content of macro-, micro-elements and trace elements in the egg albumen. (mg/kg wet wt).

Element	Group-C	Group-E	SEM	*p*-Value
Macroelements				
S	1984 ^b^	2114 ^a^	33.7	0.045 ^†^
Na	1858 ^A^	1726 ^B^	25.2	0.001 ^†^
K	1610	1670	27.7	0.306 ^†^
P	125.2	122.2	3.17	0.664 ^†^
Mg	123.4	121.9	2.53	0.800 ^†^
Ca	73.0	72.4	3.03	0.927 ^†^
Microelements				
Si	6.84	6.86	0.541	0.986 ^†^
Al	1.09 ^B^	2.08 ^A^	0.170	0.001 ^†^
Fe	0.402	0.320	0.0501	0.431 ^†^
Cu	0.188	0.174	0.0112	0.250 ^‡^
Zn	0.171	0.107	0.0221	0.143 ^‡^
Sr	0.154	0.140	0.0101	0.250 ^‡^
I	0.050	0.053	0.0012	0.417 ^†^
Ba	0.012	0.013	0.0021	0.911 ^†^
Mn	0.011 ^a^	0.007 ^b^	0.0010	0.031 ^†^
Trace elements				
Rb	1.41 ^A^	1.08 ^B^	0.050	0.001 ^†^
Ni	0.259 ^a^	0.520 ^b^	0.0403	0.012 ^‡^
Ti	0.172	0.140	0.0101	0.245 ^‡^
Se	0.137	0.127	0.0220	0.632 ^†^
Bi	0.120 ^A^	0.153 ^B^	0.0060	0.001 ^†^
V	0.083	0.121	0.0203	0.723 ^‡^
Ag	0.082	0.076	0.0020	0.111 ^†^
Cr	0.063 ^A^	0.033 ^B^	0.0061	0.003 ^†^
As	0.047	0.048	0.0052	0.296 ^‡^
Mo	0.021	0.023	0.0010	0.911 ^†^
Ga	0.016	0.017	0.0007	0.368 ^†^
Zr	0.015	0.018	0.0021	0.834 ^‡^
Pb	0.011	0.019	0.0070	0.834 ^‡^
Sb	0.0086 ^A^	0.0060 ^B^	0.00040	0.001 ^†^
Li	0.0082	0.0089	0.00200	0.713 ^‡^
Co	0.0056 ^a^	0.0067 ^b^	0.00031	0.017 ^†^
Be	0.0045	0.0043	0.00062	0.853 ^†^
Y	0.0041	0.0046	0.00010	0.155 ^†^
Sn	0.0042	0.0037	0.00021	0.071 ^†^
Cd	0.0015 ^a^	0.0012 ^b^	0.00007	0.013 ^†^
W	0.0010 ^a^	0.0015 ^b^	0.00029	0.036 ^‡^
Tl	0.0008 ^A^	0.0012 ^B^	0.00030	0.001 ^†^
Hg	0.0005	0.0004	0.00011	0.058 ^†^

SEM—standard error of mean; ^a,b^—significance of differences on the level *p* < 0.05; ^A,B^—significance of differences on the level *p* < 0.01; ^†^—based on t-test; ^‡^—based on Mann−Whitney U test.

**Table 4:** The content of macro-, micro-elements and trace elements in the egg yolk (mg/kg wet wt).

Element	Group-C	Group-E	SEM	*p*-Value
Macroelements				
P	5858	6032	90.6	0.367 ^†^
S	1864	1840	15.3	0.466 ^†^
Ca	1358	1330	36.8	0.727 ^†^
K	1158 ^A^	1320 ^B^	31.8	0.002 ^†^
Na	593.8 ^a^	525.0 ^b^	17.20	0.035 ^†^
Mg	138.8	130.1	2.98	0.143 ^‡^
Microelements				
Fe	55.6 ^A^	81.8 ^B^	4.68	0.001 ^†^
Zn	39.6	40.2	0.97	0.777 ^†^
Si	7.58	6.81	0.290	0.201 ^†^
Al	3.34	1.97	0.611	0.292 ^†^
Ba	2.20	2.66	0.171	0.192 ^†^
Cu	1.40	1.46	0.080	0.403 ^‡^
I	1.39 ^a^	2.20 ^b^	0.162	0.012 ^‡^
Mn	0.688	0.658	0.0310	0.629 ^†^
Sr	0.136 ^A^	0.328 ^B^	0.3231	0.001 ^†^
Trace elements				
Rb	0.864	0.641	0.0401	0.121 ^†^
Sn	0.057 ^A^	0.043 ^B^	0.0070	0.001 ^†^
Cr	0.349	0.458	0.0602	0.406 ^†^
Se	0.328	0.422	0.0200	0.095 ^‡^
Ni	0.197	0.276	0.0803	0.676 ^‡^
V	0.166	0.187	0.0201	0.713 ^‡^
Ga	0.110	0.121	0.0007	0.490 ^†^
Pb	0.105	0.118	0.0083	0.467 ^†^
Mo	0.168	0.160	0.0079	0.626 ^†^
Ag	0.080	0.082	0.0093	0.907 ^†^
Ti	0.038 ^A^	0.042 ^B^	0.0008	0.005 ^†^
Zr	0.027	0.021	0.0040	0.550 ^†^
Li	0.025 ^A^	0.033 ^B^	0.0011	0.001 ^†^
Be	0.024	0.015	0.0022	0.111 ^‡^
Co	0.021	0.016	0.0030	0.834 ^‡^
Bi	0.014 ^A^	0.025 ^B^	0.0021	0.001 ^†^
As	0.009	0.005	0.0020	0.676 ^‡^
Sb	0.0063 ^A^	0.0077 ^B^	0.00031	0.003 ^†^
Y	0.0061 ^A^	0.0150 ^B^	0.00110	0.001 ^†^
Tl	0.0060 ^A^	0.0088 ^B^	0.00044	0.001 ^†^
Hg	0.0049 ^A^	0.0026 ^B^	0.00121	0.001 ^†^
W	0.0030 ^A^	0.0045 ^B^	0.00031	0.002 ^†^
Cd	0.0012	0.0013	0.00053	0.834 ^‡^

SEM—standard error of mean; ^a,b^—significance of differences on the level *p* < 0.05; ^A,B^—significance of differences on the level *p* < 0.01; ^†^—based on t-test; ^‡^—based on Mann−Whitney U test.
